# Type A Aortic Dissection in a Previously Healthy Pregnant Patient: A Challenging Dilemma, Case Report, and Literature Review

**DOI:** 10.1155/cric/6971509

**Published:** 2024-12-26

**Authors:** Elham Bateni, Maryam Dehghan, Zeinab Ahmadikia

**Affiliations:** ^1^Obstetrics and Gynecology Department, Isfahan University of Medical Sciences, Isfahan, Iran; ^2^Internal Medicine Department, Isfahan University of Medical Sciences, Isfahan, Iran

**Keywords:** aortic dissection, cardiothoracic surgery, pregnancy

## Abstract

Aortic dissection (AoD) is a rare fatal condition in which tearing in the intima causes a false channel in the aorta and can lead to rupture. AoD is classified as the DeBakey classification (Types I, II, III) and Stanford classification (Types A and B). Women with underlying risk factors such as hypertension, smoking, bicuspid aortic valve, and connective tissue disorders are at risk for pregnancy-related AoD. These risk factors may not be recognized until the AoD occurs during pregnancy. We describe an acute incidence of type A AoD in the second trimester of pregnancy. A multiparous woman with no previously known risk factor presented with nonspecific chest pain. She was found to have AoD and underwent successful surgical intervention. This case demonstrates the importance of vigilance in the evaluation of pregnant women with new cardiopulmonary symptoms. A multidisciplinary approach can save the mother and the fetus.

**Summary**

• Women with underlying risk factors are at risk for pregnancy–related aortic dissection (AoD).
• We describe an acute incidence of AoD in pregnancy managed successfully by surgical intervention.

## 1. Introduction

AoD is an uncommon fatal condition in which tearing in the intima, the inner layer of the aorta, extends to the medial layer and causes a false channel in the aorta and can lead to aortic rupture [1]. AoD is classified as the DeBakey classification (Types I, II, III) and Stanford classification (Types A, B). Type I refers to dissections that propagate from the ascending aorta and extend to the aortic arch and, commonly, beyond the arch distally. Type II refers to dissections confined to the ascending portion of the aorta. Type III dissections are limited to the descending aorta. Type III also includes the dissections that start in the descending aorta and extend proximally to the arch and ascending aorta. Stanford Type A includes dissections that involve the ascending aorta, arch, and descending thoracic aorta. Stanford Type B refers to dissections involving only the descending aorta.

Type A dissection is associated with significantly higher morbidity and mortality and requires emergency surgical intervention through a sternotomy, whereas Type B dissection is primarily managed with medical therapy unless it is complicated [2]. According to population-based studies, the annual incidence of AoD is 2.9–4.7 cases per 100,000 [3, 4]. The mortality rate is about 30% and almost occurs in the first 10 days [5]. Hypertension, smoking, bicuspid aortic valve, and genetic disorders such as Marfan syndrome (MFS), Turner syndrome, Noonan syndrome, Loeys–Dietz syndrome, and Ehlers–Danlos syndrome are risk factors for AoD [5, 6]. However, many women with pregnancy-related AoD are not known to be at risk before pregnancy [7, 8]. In this article, we describe an acute incidence of AoD in the second trimester of pregnancy.

## 2. Case Presentation

**Figure 1:**
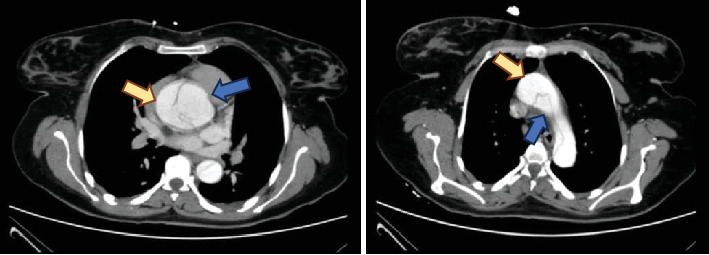
Dissection of ascending aorta with dissection flap originating at the level of the aortic root (blue arrows indicate the true lumen, and yellow arrows indicate the false lumen).

A 24-year-old G6P4Ab1 pregnant woman at a gestational age of 25 weeks was admitted with sudden onset chest pain and 2-day-long fatigue. She did not have appropriate prenatal care, and her only medical condition was lens dislocation requiring ophthalmic intervention in the past. She has stable vital signs on presentation, but blood pressure was 120/65 mmHg in the left arm and 90/60 mmHg in the right arm. She was noted to have a diastolic murmur on the exam. There was no pathologic finding in ECG, but transthoracic echocardiography revealed mild mitral valve prolapse, mild to mod aortic valve regurgitation, and a dissection flap at the sinuses of Valsalva. Abdominopelvic computed tomography angiography (CTA) confirmed dissection of the ascending aorta with a dissection flap originating at the level of the aortic root with the extension of the dissection to the left common carotid artery and left subclavian artery (Figure 1). There was no pathologic finding in other organs including pulmonary vessels and abdominal aorta. Laboratory tests including CBC, coagulation profile, and renal-hepatic function were normal. Fetal biometric sonography showed a male AGA (appropriated for gestational age) fetus. The uterine tocogram showed no contraction, and fetal heart rate (FHR) monitoring was reassuring. According to the diagnosis of Type A AoD, the mother was transferred to a tertiary cardiac center for surgical intervention. The patient underwent cardiopulmonary bypass (CPB) with continuous FHR monitoring. During surgery, aortic root dissection with extension to aortic arch with extensive hematoma was seen. Due to the involvement of the noncoronary cusp of the trileaflet aortic valve and the upper part of the right and left coronary arteries, the patient underwent the Bentall procedure (mechanical valve replacement, aortic root replacement, and coronary artery reimplantation). After surgery, FHR monitoring was reassuring, and considering that there was no uterine contraction, there was no need to administer a tocolytic agent and corticosteroid. Post-op NST (a nonstress test that measures the FHR in response to movement and contraction) was reassuring. Mother was discharged 12 days later with aspirin and warfarin, considering the high risk of thrombosis with metal valve and passing the first high-risk trimester of pregnancy in terms of teratogenicity. Although high blood pressure was not detected in our patient, metoprolol (50 mg BID) and amlodipine (5 mg daily) were also administered to keep mean blood pressure under 100 mHg to prevent recurrence of rupture. She was followed up in our obstetric clinic for up to 34 weeks. During this period, fetal echocardiography was performed, which was normal. In sequential biometric sonography, fetal growth retardation was reported (abdominal circumference < 3%), and considering the risk of emergency surgery, warfarin was changed to a therapeutic dose of enoxaparin. She underwent emergency cesarean section in 34 + 5 due to fetal distress (Biophysical Profile Score: 4/8 and nonreactive NST that was refractory to oxygen support, fluid compensation, and position change). A male baby with Apgar scores 6 and 9 at 1 and 5 min was delivered and was supported only with oxygen in the first minutes of delivery, and there was no need for admission at NICU. There was no pathologic finding in the amniotic fluid and umbilical cord, but there was calcification in the placenta which was explained by fetal growth retardation. There were no unexpectable hemorrhagic events during cesarean section and postpartum period as well as thrombotic events, hematoma, or infection for both the mother and the newborn. The mother and neonate were discharged in good condition, and the patient did not experience any complications in her monthly, 6-month, and annual follow-up.

## 3. Discussion

We presented a pregnant woman at 26 weeks of gestation with acute chest pain who underwent open heart surgery during pregnancy for acute Type A AoD. Lingchao Liu et all presented three cases that were complicated by Type A AoD in the third trimester of pregnancy and managed successfully by surgical repair of the aorta and cesarean delivery, while we continued the pregnancy after aortic repair based on the age of the pregnancy [9].

The differential diagnoses of chest pain in pregnant women include musculoskeletal pain, gastrointestinal reflux disease, pulmonary emboli, and cardiac diseases such as AoD and acute coronary syndrome. Moreover, life-threatening cardiac diseases require prompt diagnosis and management to save both mother and fetus [10].

AoD, a rare and life-threatening condition caused by a tear in the intimal layer of the aorta, can be classified into two Stanford categories, Type A and Type B [11]. Diagnostic tools for AoD, including echocardiography and CT angiography, both confirmed the diagnosis of Type A AoD in our patient.

Although AoD in pregnancy is rare, occurring in only 0.0004% of pregnancies, hemodynamic stress and alterations in connective tissue related to hormonal changes may be responsible for increased susceptibility to AoD during pregnancy [8, 12], as 1/2 cases of AoD in young women have been reported during pregnancy. Risks may be higher when conditions such as aortic valve abnormalities, high blood pressure, or connective tissue disorders such as MFS are present [12]. Major and minor criteria of MFS are based on abnormalities in skeletal, ocular, cardiovascular, pulmonary, and skin systems and genetic studies [13]. The only finding in our patient was lens dislocation requiring surgery a few years ago as a major criterion and mild mitral valve prolapse as a minor criterion. We could not evaluate the aortic valve, because the first echocardiography in our patient was done after AoD. The diagnosis of MFS may be challenging and often remains undiagnosed before pregnancy and is recognized only after life-threatening complications occur in pregnancy [13, 14]. Our patient did not undergo genetic testing, so she cannot be considered a definite MFS case. The patient with Turner syndrome also can increasingly become pregnant due to the development of assisted reproductive technology including egg donation. Whereas, AoD commonly occurs in these pregnant women [15]. The history and physical examination of our patients revealed no evidence to raise suspicion about Turner syndrome.

Women with pre-existing aortic disease and evidence of prepregnancy aortic dilatation, especially in the presence of connective tissue disorders, are indicated for elective cardiac surgery before pregnancy [16]. If acute AoD occurs during pregnancy, it is a potentially fatal complication for the mother and fetus. [17] Although our patient was in the late second trimester, AoD in most cases occurs in the third trimester and postpartum [8]. Maternal outcomes related to AoD include mortality in 6.8%–24.6% of cases, and perinatal complications include fetal loss, stillbirth, and neurological complications in 10%–20%. [17] In our case, even though the fetus was saved during surgery, he showed mild growth retardation which can be considered as a complication of open cardiac surgery during pregnancy.

Although Stanford Types A AoD, the most common category in pregnant women, is a surgical emergency, the risk of fetal death up to 30% associated with CPB must be considered. It is recommended to deliver the viable fetus first and then repair the open aorta. If the fetus is nonviable, open aortic repair with CPB is recommended during pregnancy after counseling about the risk of fetal loss [18]. According to several different studies, patients with a gestational age of less than 28 weeks are candidates for aortic repair while the fetus is in utero, which is what we did in our patient. However, patients past 32 weeks of gestation would primarily require cesarean section followed by aortic repair. Pregnant women between gestational ages of 28 and 32 weeks, would have to be carefully evaluated for the appropriate delivery method depending on fetal condition [11, 14].

Initial medical management involving the control of blood pressure using antihypertensive medications is recommended for pregnant women presenting with Stanford Type B AoD. The failure of medical management is followed by surgical treatment [19, 20].

When a woman undergoes cardiac surgery with CPB during pregnancy, FHR should be monitored continuously, the duration of CPB should be reduced, and the perfusion flow and pressure of CPB should be improved during the operation [13]. In our case, the operation time was about 6 h. The perfusion was performed with high flow (between 3 and 4 L/min/m^2^), high perfusion pressure (> 70 mmHg), and high hematocrit (> 20%), and the FHR was monitored by external FHR monitoring throughout the operation. Fetal death most often occurs during the cooling and rewarming phases of CPB. Thus, the risk of fetal death can be greatly reduced by controlling the temperature changes during the diversion [13]. We tried to keep the temperature at about 34°C. Our strategy saved both the fetus and mother in this case.

## 4. Conclusion

Undiagnosed aortic valvopathies and silent collagen vascular diseases may lead to acute AoD in an asymptomatic patient during pregnancy. Our patient presented with nonspecific chest pain without any previously proven risk factors. This case demonstrates the importance of vigilance in the evaluation of pregnant women with new cardiopulmonary symptoms that could be related to one of several life-threatening causes such as AoD that requires a multidisciplinary approach to save the mother and fetus.

## Data Availability

All data underlying the results are available as part of the article, and no additional source data are required.
